# Ebola Policies That Hinder Epidemic Response by Limiting Scientific Discourse

**DOI:** 10.4269/ajtmh.14-0803

**Published:** 2015-02-04

**Authors:** Ramin Asgary, Julie A. Pavlin, Jonathan A. Ripp, Richard Reithinger, Christina S. Polyak

**Affiliations:** Departments of Population Health and Medicine, New York University School of Medicine, New York, New York; Armed Forces Surveillance Center, Silver Spring, Maryland; Department of Medicine, Icahn School of Medicine at Mount Sinai, New York, New York; Global Health Division, International Development Group, Research Triangle Institute, Washington, DC; Military HIV Research Program, Walter Reed Army Institute of Research, Bethesda, Maryland

## Abstract

There is an unprecedented epidemic of Ebola virus disease (EVD) in west Africa. There has been a strong response from dedicated health professionals. However, there have also been irrational and fear-based responses that have contributed to misallocation of resources, stigma, and deincentivizing volunteers to combat Ebola at its source. Recently, the State of Louisiana Department of Health and Hospitals issued a ban on those coming from affected countries wishing to attend the annual meetings of American Society of Tropical Medicine and Hygiene and the American Public Health Association, both of which were held in New Orleans. We argue against such policies, question evidence and motivations, and discuss their practical and ethical implications in hampering effective responses to EVD by the scientific community. We aim to shed light on this issue and its implications for the future of public health interventions, reflect on the responsibility of health providers and professional societies as advocates for patients and the public health, and call for health professionals and societies to work to challenge inappropriate political responses to public health crises.

On October 28, 2014, 5 days before the annual meeting of the American Society of Tropical Medicine and Hygiene (ASTMH) in New Orleans, the Louisiana Department of Health and Hospitals (DOHH) in conjunction with the Governor's Office for Homeland Security and Emergency Preparedness announced to all ASTMH attendees that “individuals who traveled to and returned from the countries of Sierra Leone, Liberia or Guinea in the past 21 days, or have had contact with a known EVD [Ebola virus disease] patient in that time period, should NOT travel to New Orleans to attend the conference. Given that conference participants with a travel and exposure history for EVD are recommended not to participate in large group settings (such as this conference) or to utilize public transport, we see no utility in you traveling to New Orleans to simply be confined to your room.” Furthermore, the letter stated that “from a medical perspective, asymptomatic individuals are not at risk of exposing others; however, the State is committed to preventing any unnecessary exposure of Ebola to the general public. In Louisiana, we love to welcome visitors, but we must balance that hospitality with the protection of Louisiana residents and other visitors.”^1^

While acknowledging recommendations of the Centers for Disease Control and Prevention (CDC) that asymptomatic individuals are not a risk to others, the statement went beyond the CDC guidelines and implied a potential threat from conference attendees, even those without exposure to EVD, based solely on travel history to countries affected by the epidemic. We believe the DOHH should appreciate the negative ramifications of unscientifically based travel bans and quarantine policies and rather, follow evidence-based guidelines to protect the public and avoid legitimizing irrational responses caused by fear.

Ironically, the ASTMH is the pre-eminent professional society in tropical medicine, and the annual meeting of the society is an ideal place to share scientific advances in response to EVD, an interchange that benefits both the United States and all countries facing the current epidemic. Prospective conference attendees who are actively engaged in the EVD response were prepared to share their experiences in scientific sessions, but some could not attend. Numerous attendees from west Africa, including countries not directly affected by EVD, may have been afraid to attend because of not knowing whether they would be turned away on arrival. Moreover, the DOHH reiterated their travel ban for attendees of the annual conference of the American Public Health Association held November 15–19 in New Orleans.

Ebola virus causes a deadly disease, and it typically occurs in places that have underresourced and overwhelmed health systems; whereas prior outbreaks have been small and contained, the current outbreak in west Africa is of unprecedented scale.^2^ In September of 2014, the World Health Organization declared the current Ebola virus disease (EVD) outbreak a major threat to global health and security and requested that all global health organizations and supporting countries maximize their efforts to combat the disease at its source.^3^ Sporadic cases in high-income countries have occurred connected to this outbreak. Because the virus is known to be transmitted by physical contact, the risk of an EVD epidemic in countries with well-equipped public health and medical systems is small. In the past, limited quarantine procedures and travel bans have been enacted for highly contagious diseases, such as Severe Acute Respiratory Syndrome (SARS). However, considering limited transmission of Ebola virus to casual contacts, there is no evidence to suggest that these strategies are needed to control EVD. On the contrary, there are detrimental consequences of inappropriately combating the outbreak in this manner. For one, health professionals who are desperately needed to combat the disease at its source are disincentivized to risk their own health.^4^ Current fear-fueled policies issued by several states in the United States are causing significant stigma toward health workers, their families, and the organizations that respond to EVD epidemics; they also marginalize people of west African descent who live in the United States and have not had any exposure to EVD.^5^ This would not be the first time that irrational reactions hampered scientific advancement and harmed patients—during the early Acquired Immune Deficiency Syndrome (AIDS) epidemic, at-risk populations were similarly marginalized.

Unfounded policies, such as the Louisiana DOHH response, also have the potential to encourage potentially exposed individuals to travel outside of monitored routes, deny their exposure, and avoid diagnosis and isolation when symptomatic. Instead, the DOHH should adopt policies based on evidence, such as the established protocols of Médecins Sans Frontières and the CDC,^6^ which advise monitoring returned asymptomatic health workers. These are effective and should continue to be the basis for a response to EVD in the United States.

In the case of the current EVD epidemic and other public health crises, there is a need for greater advocacy on the part of health professionals and academic and professional institutions. Beyond the responsibility of providers to care for individual patients, health professionals should raise awareness about the public health implications of inappropriate responses and policies to public health crises. The medical community should unite and attack inappropriate policies to better protect our patients and their communities. Broader advocacy at the national level and within professional societies is needed to eschew fear-induced and political decisions and maintain evidence-based, neutral, and destigmatizing responses. Such actions would serve to refocus discussion on the evidence and show solidarity on the part of health professionals with the affected population as well as the heroic providers who have chosen to combat Ebola at its source.
